# Ddb1 Is Essential for the Expansion of CD4^+^ Helper T Cells by Regulating Cell Cycle Progression and Cell Death

**DOI:** 10.3389/fimmu.2021.722273

**Published:** 2021-08-30

**Authors:** Lingtao Yang, Wei Chen, Li Li, Yueyue Xiao, Shilin Fan, Quan Zhang, Tian Xia, Mengjie Li, Yazhen Hong, Tongjin Zhao, Qiyuan Li, Wen-Hsien Liu, Nengming Xiao

**Affiliations:** ^1^State Key Laboratory of Cellular Stress Biology, Innovation Center for Cell Signaling Network, School of Life Sciences, Xiamen University, Xiamen, China; ^2^School of Medicine, Xiamen University, Xiamen, China

**Keywords:** Ddb1, follicular helper T cells, humoral immunity, DNA damage response, T cell differentiation, G2/M arrest, Th1

## Abstract

Follicular helper T (T_FH_) cells are specialized CD4^+^ helper T cells that provide help to B cells in humoral immunity. However, the molecular mechanism underlying generation of T_FH_ cells is incompletely understood. Here, we reported that Damage-specific DNA binding protein 1 (Ddb1) was required for expansion of CD4^+^ helper T cells including T_FH_ and Th1 cells, germinal center response, and antibody response to acute viral infection. *Ddb1* deficiency in activated CD4^+^ T cells resulted in cell cycle arrest at G2-M phase and increased cell death, due to accumulation of DNA damage and hyperactivation of ATM/ATR-Chk1 signaling. Moreover, mice with deletion of both *Cul4a* and *Cul4b* in activated CD4^+^ T cells phenocopied *Ddb1*-deficient mice, suggesting that E3 ligase-dependent function of Ddb1 was crucial for genome maintenance and helper T-cell generation. Therefore, our results indicate that Ddb1 is an essential positive regulator in the expansion of CD4^+^ helper T cells.

## Introduction

CD4^+^ T cells have a vital role in adaptive immune responses and can differentiate into functionally distinct subsets, including effector helper T cells (type 1 helper T cells (Th1), Th2, Th17, T_FH_) or regulatory T cells (Treg), triggered by environmental cytokine milieu. Among them, T_FH_ cells provide mutual help signals to B cells in germinal center (GC), where B cells undergo somatic hypermutation and affinity maturation and differentiate into memory B cells and long-lived plasma cells (1). T_FH_ cells are characterized by surface expression of chemokine receptor CXCR5, inducible costimulator (ICOS), and programmed cell death protein 1 (PD-1) (2, 3). The transcription repressor Bcl-6 has been identified as a master transcription factor for T_FH_ cells (4–6). The differentiation of T_FH_ cells consists of multistage, multifactorial, spatiotemporally regulated processes, and is initiated by ICOS receptor engagement, driven by network of transcription factors and influenced by several cytokines (2, 3). Thus, better understanding of the differentiation and function of T_FH_ cells is important for development of vaccine or therapeutic approaches to boost immune responses against pathogen infection.

Current understanding suggests that activation-induced T-cell cycle progression and T-cell death are crucial for T-cell mediated immune responses. Upon antigen stimulation, naïve CD4^+^ T cells grow in size followed by cell cycle entry and rapid expansion and subsequently differentiate into distinct subsets of effector CD4^+^ T cells including T_FH_ cells in various cytokine microenvironment (7, 8). When the antigens were diminished, most of the effector CD4^+^ T cells undergo cell death and the others differentiate into memory CD4^+^ T cells (9, 10). T-cell expansion and T-cell death were tightly controlled by multiple regulatory checkpoints. One of these is G2/M DNA damage checkpoint that prevents rapidly proliferating cells from entering mitosis (M-phase) with unrepaired DNA damage induced by DNA replication stress. Accumulation of unrepaired DNA damage could induce cell cycle arrest or apoptosis (11, 12). However, the mechanism that allows T cells in clonal expansion to quickly repair DNA damage to avoid these cell fates remains unclear.

Damage DNA binding protein 1 (Ddb1) is originally identified as a protein heterodimerizes with Ddb2 and a subunit of the UV-DDB complex which recognizes the UV-induced DNA lesions in the nucleotide excision repair (NER) pathway (13). Thus, Ddb1 is required for preventing the replication of damaged DNA and also for DNA repair and the maintenance of genome integrity (14–17). Ddb1 is a component of Cullin 4-RING ubiquitin E3-ligases (CRL4) and functions as an adaptor protein to link Cullin 4 (Cul4) and CUL4-associated factors (DCAFs) to form Cul4-Ddb1-DCAFs E3 ligase complex that targets various substrates for ubiquitination, including Cdt1 (18, 19), p21 (20, 21), Chk1 (22), and Tsc2 (23). Beyond that, DDB1 can also directly stimulate gene transcription independent of E3 ligase-mediated processes, indicating Ddb1 exerts its diverse cellular function in both E3 ligase-dependent and -independent manner (24).

Here, we show that Ddb1 is essential for expansion of T_FH_ and Th1 cells, GC response, and antiviral antibody response during acute viral infection. We further revealed that Ddb1 ablation led to accumulation of DNA damage, cell cycle arrest, and increase of cell death, thereby resulting in the defect of T_FH_ and Th1 cell expansion. Ultimately, we identified that the function of Cul4-Ddb1-DCAFs E3 ligase complexes was crucial for genome maintenance and CD4^+^ helper T-cell generation. Our study demonstrates that Ddb1 is an essential positive regulator of CD4^+^ helper T-cell expansion, including T_FH_ and Th1 cells.

## Material and Methods

### Mice

*Ddb1*^fl/fl^ mice were a generous gift from Yong Cang at Shanghai Tech University (25). *Trp53* ^fl/fl^ mice were provided by Dawang Zhou at Xiamen University. *Cul4a* ^fl/fl^ mice (Cul4a^tm1a(EUCOMM)Hmgu^, #10587) were purchased from EUCOMM. SMARTA mice were provided by Shane Crotty at La Jolla institute for Immunology. OX40-cre mice (#012839), Rag2^-/-^ mice (#008449), C57BL/6J mice (#000664), and B6.SJL mice (#002014) were originally from the Jackson Laboratory.

### Antibodies and Reagents

Fluorescence-conjugated antibodies for CD4(Cat # 48-0042-82), CD8(Cat # 47-0081-82), CD44(Cat # 11-0441-85), CD45.1(Cat # 47-0453-82), CD45.2(Cat # 48-0454-82), Streptavidin(Cat # 25-4317-82), IgD(Cat # 48-5993-82), and Ki67(Cat # 25-5698-82) were purchased from eBioscience; CXCR5(Cat # 551961), CD138(Cat # 553713), IgG2α(Cat # 553894), PD1(Cat # 562671), Bcl-6(Cat # 561525), Fas(Cat # 554258), GL7(Cat # 553666), and H2A.X(pS139)(Cat # 564719) were purchased from BD Biosciences; B220(Cat # 103212), SLAM(Cat # 115904), IFNγ(Cat # 505810), and FITC Annexin V Apoptosis detection kit with 7-AAD(Cat # 640922) were purchased from Biolegend.

Anti-Ddb1 (cat #11380-1-AP), anti-β-Actin (cat #66009-1-Ig), and anti-ATR (cat #19787-1-AP) were purchased from Proteintech; anti-p53 (cat #2524), phosphor-Chk1 (S345) (cat #2348T), anti-cleaved-caspase8 (Asp387) (cat #8592S), anti-caspase8 (cat #4790T), anti-cleaved-caspase3 (Asp175) (cat #9664S), and anti-caspase3 (cat #9662) were purchased from Cell Signaling technology (CST); anti-Prkdc(cat #SC-390849) was purchased from SantaCruz; anti-ATM (cat #A19650) and anti-Chk1 (cat #A7653) were purchased from ABclonal Technology.

### Flow Cytometry and Cell Sorting

Lymphocytes were isolated from the thymus, spleen, and lymph nodes of age- and gender-matched mice of 6–8 weeks of age. Single-cell suspensions of lymphocytes were prepared by mashing of tissues through a cell strainer and were treated with red-blood-cell-lysis buffer. Surfaces of cells were stained with fluorochrome-conjugated antibodies in flow cytometry buffer (0.5% BSA and 0.05% NaN3 in PBS). A three-step CXCR5 staining was performed with purified anti-CXCR5 (2G8; BD PharMingen), followed by biotinylated goat antibody to rat IgG (112-065-167; Jackson Immunoresearch) and then PECy7-labeled streptavidin (25-4317-82; eBioscience), with each staining step done at 4°C in CXCR5 staining buffer (0.5% BSA, 2% FCS, and 2% normal mouse serum in PBS).

Intracellular cytokines were stained after stimulation of cells for 4 h with 50 ng/ml PMA (phorbol 12-myristate 13-acetate; Sigma-Aldrich) and 1 μg/ml ionomycin (Sigma-Aldrich) in the presence of GolgiStop. Cells were incubated with antibodies to cell surface markers (all identified above), and then were fixed and permeabilized with Cytofix/Cytoperm Buffer (51-2090KZ; BD Biosciences). Cells were then stained with antibodies to cytokines (all identified above). Intracellular Bcl-6 or Foxp3 was stained after cell-surface staining. Samples were fixed and permeabilized with Foxp3 staining buffer according to the manufacturer’s manual (00-5523; eBioscience). Samples were incubated for 45–60 min at 4°C with Fixation/Permeabilization buffer and washed with 1× permeabilization buffer. Samples were incubated for another 45–60 min with fluorochrome-conjugated monoclonal antibody to Bcl-6 (K112-91; BD Biosciences) or Foxp3 (FJK-16s; eBioscience) in permeabilization buffer. For cell sorting, cell suspensions were stained with fluorochrome-conjugated antibodies in sorting buffer (PBS supplemented with 1 mM EDTA, 25 mM HEPES, 1% FBS). Stained cells were either analyzed on the BD LSRFortessa or sorted on the BD Aria Fusion cell sorter. Flow cytometry was analyzed with FlowJo software.

### ELISA

Concentrations of anti-LCMV IgG and IgG3 in serum of mice infected with LCMV ARMSTRONG were quantified by ELISA. Lysates of cells infected with LCMV Armstrong had been inactivated by ultraviolet irradiation as the capture antigen. Ninety-six-well MaxiSorp microtiter plates (ThermoFisher Scientific: cat #439454) were coated overnight lysates of cells infected with LCMV inactivated by ultraviolet irradiation, in PBS. After incubation of sample serum, plates were incubated with biotin-conjugated goat antibody to mouse IgG (Southern Biotech, cat #1030-08), IgG1 (Southern Biotech, cat #1070-08), IgG2a (Southern Biotech, cat #1080-08), IgG2b (Southern Biotech, cat #1090-08), and IgG3 (Southern Biotech, cat #1100-08), followed by horseradish peroxidase-conjugated streptavidin (Southern Biotech, cat #7100-05) and then tetramethyl-benzidine substrate solution (Bio-Rad, cat #172-1068).

### Bone Marrow Chimera

Bone marrow cells were isolated from age- and gender-matched Ddb1^fl/fl^; OX40-Cre mice (CD45.1^+^ CD45.2^+^) and WT mice (CD45.2^+^). Bone marrow cells of different origins were mixed at the ratio of 1:1 and transferred into irradiated *Rag2^-^* ^/-^ mice to generate chimeric mice. Effector CD4^+^ T-cell population and function analysis were performed at 8–9 weeks post cell transfer.

### LCMV Infection and T-Cell Adoptive Transfer Assay

LCMV Armstrong strain was propagated in BHK-21 cells. Viral stocks were diluted in plain RPMI. Each mouse was infected with 2 × 10^5^ plaque-forming units of LCMV Armstrong for experiment at day 8 by bilateral intraperitoneal injection.

For T-cell adoptive transfer assay, naïve CD4^+^ T cells were purified from Ddb1^fl/fl^; OX40-Cre; SMARTA mice (CD45.1^+^) and WT SMARTA mice (CD45.1^+^ CD45.2^+^). Naïve CD4^+^ T cells of different origins were mixed at the ratio of 1:1 and transferred into WT mice (CD45.2^+^). Twenty-four hours post transfer, recipient mice were injected intraperitoneal with 1 × 10^6^, 5 × 10^5^, and 2 × 10^5^ plaque-forming units of LCMV Armstrong for experiments at days 2, 3, and 8, respectively.

### Cell Cycle Analysis

*In vitro* activated CD4^+^ T cells were cultured with 10 µM EdU for 2 h prior to harvest, stained with EdU staining kit as per manufacture’s protocol (Beyotime #C0081L), and analyzed by flow-cytometry.

For *in vivo* experiments, mice were injected intraperitoneally with 200 µg EdU in PBS; 12 h later, single cell suspension of the spleen was stained with EdU staining kit as per manufacture’s protocol (Beyotime #C0081L) and analyzed by flow-cytometry.

### Immunofluorescence

After indicated treatments with mice in the figure legends, spleen and lymph nodes were fixed overnight at 4°C in PBS-buffered 1% paraformaldehyde, embedded in OCT compound (Tissue-Tek), and kept at -80°C until further processing. Eight-micrometer cross-sections through the tissue midline were prepared and permeabilized with 0.3% TritonX-100 in PBS for 10 min and blocked with 5% BSA in PBS for 30 min at room temperature. Sections were incubated with antibodies against the following proteins: Phospho-Histone H2A.X(Ser-139) from cell signaling technology (#2577); Anti-Mouse CD4 APC (eBioscience #17-0042-82); Hoechst 33342 (Beyotime #C1029); and Goat anti-Rabbit IgG(H+L)-AF488 (Thermofisher Scientific #A11034). Imaging of the sections was carried out using Leica TCS SP8 confocal laser microscopy under a ×63 oil objective.

### Western Blot

Activated CD4^+^ T cells were collected from mice or in CD4^+^ T-cell stimulation assay, and precipitates of cells were washed once with PBS and boiled in 50 µl SDS loading buffer. Samples were separated to 8–12% SDS-PAGE, followed by electrotransfer to PVDF membranes (Millipore). Membranes were analyzed by immunoblot with appropriated antibodies, followed by horseradish peroxidase-conjugated second antibody (ABclonal #AS003 or #AS014) and development with an enhanced chemiluminescence detection system (RPN2106, RPN2232 or RPN 2235; GE healthcare).

### CD4^+^ T-Cell Stimulation Assay

Primary CD4^+^ T cells were cultured in RPMI-1640 medium supplemented with 10% FBS, HEPES (10 mM), sodium pyruvate (1 mM), β-mercaptoethanol (50 μM), penicillin, and streptomycin. Naïve CD4^+^ T cells were isolated from spleens and lymph nodes of 8-week-old WT and Ddb1^fl/fl^; OX40-cre mice, and cells were co-cultured with Antigen Presenting Cells (APC cells) in 96-well U bottom plate with soluble 1 μg/ml anti-mouse CD3 and 1 μg/ml anti-mouse CD28 antibodies. CD4^+^ T cells were activated for 0–6 days and harvested for flow cytometry analysis or western blotting.

Experiment to restore the phenotype of Ddb1 deficient activated CD4+ T cells and CD4^+^ T cells were treated with various inhibitors in CD4^+^ T-cell stimulation assay. Z-VAD (cat #HY-16658B), Z-IETD (cat #HY-101297), Ferrostatin-1 (cat # HY-100579), GSK872 (cat #HY-101872), Nec1 (cat # HY-15760), AZD7762 (cat # HY-10992), and Rabusertib (cat # HY-14720) were purchased from MedChemExpress; Nec-1s (cat #S8641) was purchased from Selleck.

### Statistical Analysis

Data analysis was processed and presented by GraphPad Prism 8.0. Statistical significance was determined by Student’s unpaired t-test. A *P* value of <0.05 was considered significant.

## Results

### Ddb1 Expression in Lymphocytes

**Figure 1:**
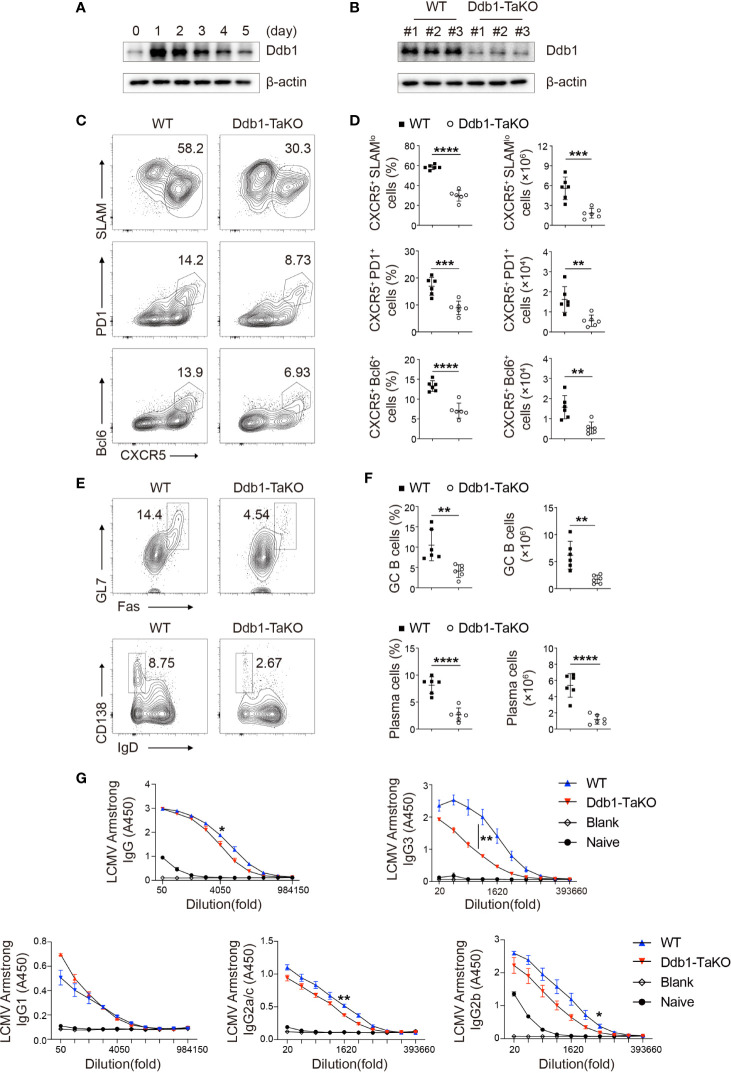
Ddb1 deficiency in activated T cells results in defective generation of T_FH_ cells. **(A)** Immunoblot analysis of Ddb1 in naïve CD4^+^ T cells activated by 3 µg/ml anti-CD3 and 3 µg/ml anti-CD28 at indicated time points. **(B)** Immunoblot analysis of Ddb1 in activated (CD44^hi^ CD62L^lo^) CD4^+^ T cells from wild type (WT) and Ddb1^fl/fl^ OX40-cre (Ddb1-TaKO) mice at day 8 after infection with LCMV Armstrong. Numbers #1, #2, and #3 represent three different pairs of mice. **(C)** Flow cytometry of activated (CD44^hi^) CD4^+^ T cells from WT and Ddb1-TaKO mice at day 8 after LCMV Armstrong infection. Numbers adjacent to outlined areas indicate percentage of CXCR5^+^ SLAM^lo^ polyclonal T_FH_ cells (top row) or CXCR5^+^ PD1^+^ GC T_FH_ cells (middle row) or CXCR5^+^ Bcl-6^+^ GC T_FH_ cells (bottom row). **(D)** Frequency among activated (CD44^hi^) CD4^+^ T cells and total number of T_FH_ cells and GC T_FH_ cells in spleen of mice as in **c** (n = 6). **(E)** Flow cytometry of total B220^+^ B cells from mice as in **(C)** Numbers adjacent to outlined areas indicate percentage of Fas^+^ GL7^+^ GC B cells (top row) or CD138^hi^ IgD^lo^ plasma cells (bottom row). **(F)** Frequency (among total B220^+^ B cells) and total number of GC B cells and plasma cells in the spleen of mice as in **(C)** (n = 6). **(G)** Enzyme-linked immunosorbent assay (ELISA) of LCMV-specific IgG, IgG1, IgG2a/c, IgG2b, and IgG3 in serum from infected mice as in **(C)** or uninfected B6 mice (n = 3 per group). Serum titers of IgG, IgG1, IgG2a/c, IgG2b, and IgG3 are presented as absorbance at 450nm (A450). Each symbol represents an individual mouse; small horizontal lines indicate the mean ( ± s.d.). *P < 0.05; **P < 0.01; ***P < 0.001; ****P < 0.0001 (Student’s unpaired t-test). Data are representative of three independent experiments (error bars, s.d.).

To evaluate the potential role of Ddb1 in immune system, we first analyzed Ddb1 expression in different mouse immune cells. According to the database of the gene-annotation portal BioGPS (raw data from GeneAtlas MOE430, gcrma), we found that mRNA of Ddb1 was expressed in various immune cells including NK cells, thymocytes, and splenocytes (**Supplementary Figure S1A**). Meanwhile, we analyzed protein levels of Ddb1 in different immune cells from mice, including thymocytes, B220^+^ B cells, and CD4^+^ and CD8^+^ T cells. Similar to its mRNA, protein of Ddb1 was also highly expressed in different immune cells (**Supplementary Figure S1B**). These findings indicated that Ddb1 might have a vital role in adaptive immunity. Recent work revealed that Ddb1 play a key regulatory role in embryonic development and B-cell development (25, 26); however, its function in regulating T-cell immunity is unknown. To clarify the correlation of Ddb1 expression and T-cell activation, purified naïve CD4^+^ T cells from wild type mice were stimulated with anti-CD3 plus anti-CD28 antibodies. We found that Ddb1 expression was robustly induced in response to TCR/CD28 engagement (**Figure 1A**), suggesting that Ddb1 has function in activated CD4^+^ T cells.

### Ddb1 Affects Peripheral T-Cell Homeostasis

To investigate the function of Ddb1 in activated CD4^+^ T cells, we crossed mice carrying loxP-flanked *Ddb1* alleles (*Ddb1* ^fl/fl^) with targeted mutant mice (Ox40-Cre) in which an internal ribosome entry site (IRES)-Cre recombinase was inserted into exon 3 of *Tnfrsf4* locus (encoding Ox40) to generate *Ddb1* ^fl/fl^ Ox40-Cre (Ddb1-TaKO) mice, which underwent deletion of Ddb1 specifically in activated CD4^+^ T and regulatory T (Treg) cells (**Figure 1B** and **Supplementary Figures S1C, D**). Since it has been reported that a small fraction of thymocytes also express Ox40 (27), we firstly investigated whether *Ddb1* deficiency in Ox40-expressing cells affected T-cell development and homeostasis. The frequencies and numbers of thymocyte populations were comparable between Ddb1-TaKO mice and their wild-type (WT) littermates (**Supplementary Figures S2A, B**), suggesting that Ddb1 in Ox40-expressing thymocytes was dispensable for T-cell development. In the periphery, although the frequencies and numbers of splenic CD4^+^ T cells were declined (**Supplementary Figures S2C, D**), the frequencies of effector memory (CD44^+^ CD62L^-^) CD4^+^ T cells and both frequencies and numbers of effector memory (CD44^+^ CD62L^-^) CD8^+^ T cells were markedly augmented in Ddb1-TaKO mice compared with that of WT counterparts (**Supplementary Figures S2E, F**). We next explored whether the activated phenotype of CD4^+^ and CD8^+^ T cells was cell-intrinsic or due to impaired Treg function. To address this issue, we generated mixed bone marrow (BM) chimeric mice by reconstituting irradiated *Rag2^-/-^* recipient mice with a mixture of congenitally marked BM cells from Ddb1-TaKO (CD45.2^+^) and WT (CD45.1^+^ CD45.2^+^) donor mice at a ratio of 1:1. Similarly, after 8 weeks of reconstitution, the thymocyte development of Ddb1-TaKO donor cells was also normal (**Supplementary Figures S3A, B, E, F**). Surprisingly, Ddb1-TaKO donor bone marrow cells generated fewer total CD4^+^ T cells and effector memory CD4^+^ T cells in spleen, while the generation of total and effector memory CD8^+^ T cells from Ddb1-TaKO donor cells was similar to that from WT donor cells (**Supplementary Figures S3C, D, G–J**). Taken together, these data suggested that the activated phenotype of CD4^+^ and CD8^+^ T cells in unimmunized Ddb1-TaKO mice was probably invoked by impaired stability or function of Treg cells, and that Ddb1 was intrinsically required for peripheral CD4^+^ T-cell homeostasis.

### *Ddb1* Deficiency Attenuates the Generation of CD4^+^ Helper T Cells

As the effector memory T cells in unimmunized mice arise from the cells that have responded to self-antigens (28), we next examined whether Ddb1 regulates T-cell response to foreign antigens. To determine the effects of Ddb1 deficiency on Th cell differentiation, we infected Ddb1-TaKO mice and their WT littermates with lymphocytic choriomeningitis virus (LCMV) Armstrong strain. At day 8 after infection, we analyzed T-cell and B-cell responses by flow cytometry. Ddb1-TaKO mice had lower proportions and cell numbers of total and effector CD4^+^ T cells, and consequently generated lower frequencies and numbers of Th1 cells (CD44^+^IFNγ^+^) than WT mice did in response to acute LCMV infection (**Supplementary Figures S4A–F**). Notably, Ddb1-TaKO mice exhibited significantly lower frequencies and absolute numbers of both total T_FH_ cells (CXCR5^+^SLAM^lo^) and GC T_FH_ cells (CXCR5^+^PD1^+^ or CXCR5^+^Bcl-6^+^) than WT mice (**Figures 1C, D**). Taken together, these data suggested that Ddb1 positively regulated the generation of CD4^+^ helper T cells, including both Th1 and T_FH_ cells.

Given that the most important function of T_FH_ cells is to initiate GC reaction and pathogen-specific antibody responses, we next examined B-cell and antibody response in Ddb1-TaKO and WT mice after LCMV Armstrong infection. As expected, we observed the percentages and cell numbers of GC B cells (FAS^+^GL7^+^) were markedly diminished in Ddb1-TaKO mice compared with WT mice at day 8 after infection. Correspondingly, the percentages and numbers of plasma cells (IgD^lo^CD138^+^) were also largely decreased in Ddb1-TaKO mice (**Figures 1E, F**). To assessed the consequences of the defective GC responses of Ddb1-TaKO mice, we measured virus-specific antibody responses by enzyme-linked immunosorbent assay (ELISA). The concentrations of LCMV-specific immunoglobulin G3 and G2b (IgG3 and IgG2b) were much lower and that of IgG2a/c and total IgG were a little lower in the sera of Ddb1-TaKO mice than in that of WT mice (**Figure 1G**). Collectively, these data suggested that Ddb1 was required for the generation of T_FH_ cells, GC B cells, and high-affinity antibody response.

### Ddb1 Intrinsically Controls CD4^+^ Helper T-Cell Expansion

**Figure 2:**
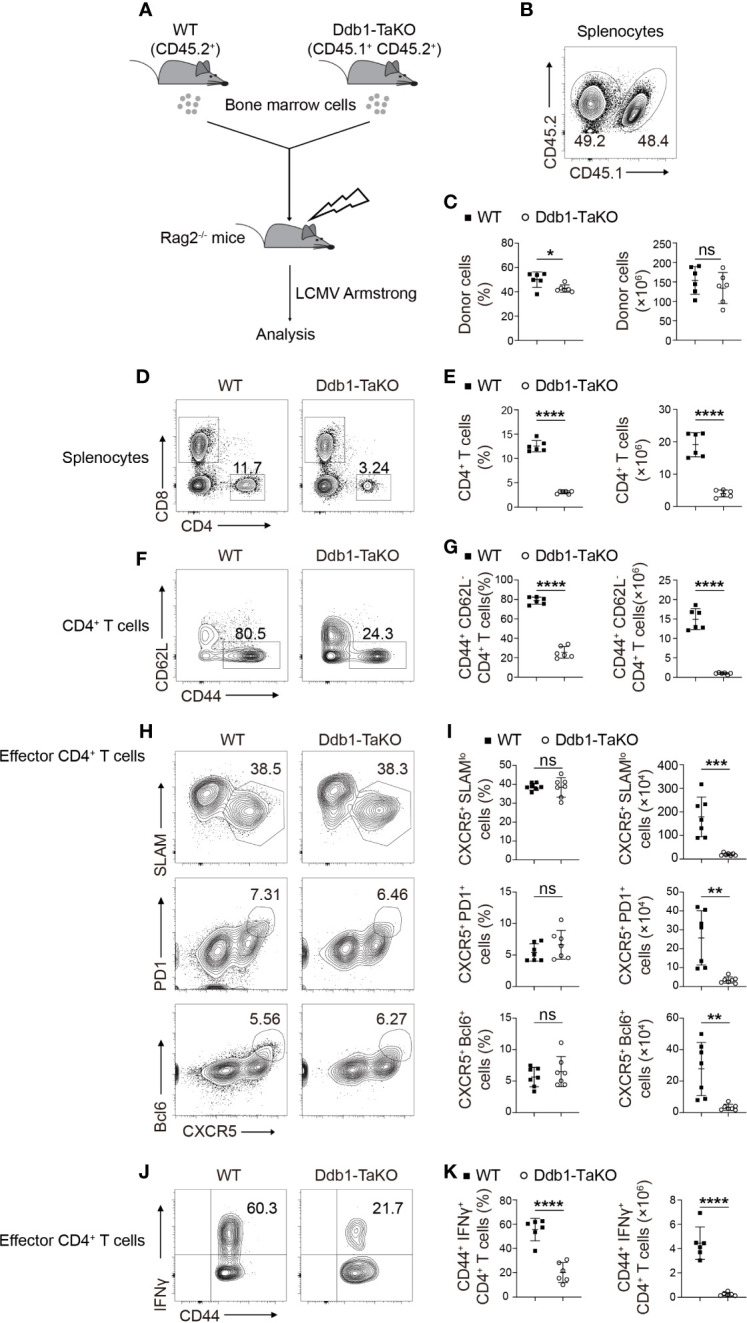
Ddb1 intrinsically controls the generation of effector CD4^+^ T cells. **(A)** Schematic diagram of mixed bone marrow (BM) chimera experiments. Donor BM cells from Ddb1-TaKO mice (CD45.1^+^ CD45.2^+^) and WT mice (CD45.2^+^) were mixed in a 1:1 ratio and transferred to irradiated Rag2^-/-^ mice. After reconstitution, the recipient mice were infected with LCMV Armstrong. **(B)** Flow cytometry of splenocytes in chimeras generated in **(A)**, assessed at day 8 after LCMV Armstrong infection. Numbers adjacent to outlined areas indicate percentage of WT or Ddb1-TaKO donor cells. **(C)** Frequency (among splenocytes) and total cell number of WT or Ddb1-TaKO donor cells in spleen of mice in **(A)** (n = 6). **(D)** Flow cytometry of WT or Ddb1-TaKO donor cells in the spleen of chimeras generated in **(A)**, assessed at day 8 after LCMV Armstrong infection. Numbers adjacent to outlined areas indicate percentage of CD4^+^ and CD8^+^ T cells. **(E)** Frequency and total number of CD4^+^ T cells in WT or Ddb1-TaKO donor cells (n = 6). **(F)** Flow cytometry of CD4^+^ T cells in WT or Ddb1-TaKO donor cells. Numbers adjacent to outlined areas indicate percentage of effector (CD44^hi^ CD62L^lo^) CD4^+^ T cells. **(G)** Frequency (among CD4^+^ T cells) and total number of effector (CD44^hi^ CD62L^lo^) CD4^+^ T cells in WT or Ddb1-TaKO donor cells (n = 6). **(H)** Flow cytometry of donor WT or Ddb1-TaKO activated (CD44^hi^) CD4^+^ T cells in the spleen of chimeras in **(A)**. Numbers adjacent to outlined areas indicate percentage of CXCR5^+^ SLAM^lo^ polyclonal T_FH_ cells (top row) or CXCR5^+^ PD1^+^ GC T_FH_ cells (middle row) or CXCR5^+^ Bcl-6^+^ GC T_FH_ cells (bottom row). **(I)** Frequency (among donor activated CD4^+^ T cells) and total cell number of polyclonal T_FH_ cells and GC T_FH_ cells in the spleen of mice in **(A)** (n = 7). **(J)** Flow cytometry of donor WT or Ddb1-TaKO activated (CD44^hi^) CD4^+^ T cells in the spleen of chimeras in **(A)**. Numbers adjacent to outlined areas indicate percentage of IFNγ^+^ CD4^+^ T cells. **(K)** Frequency (among donor CD4^+^ CD44^+^ T cells) and total number of IFNγ^+^ CD4^+^ T cells in WT or Ddb1-TaKO donor cells (n = 6). Each symbol represents an individual mouse; small horizontal lines indicate the mean ( ± s.d.). ns, not significant; *P < 0.05; **P < 0.01; ***P < 0.001; ****P < 0.0001 (Student’s unpaired t-test). Data are representative of three independent experiments (error bars, s.d.).

In Ddb1-TaKO mice, Ddb1 might also be deleted in Treg cells, which could affect immune homeostasis (**Supplementary Figures S2C–F**) and then influence the generation of effector T cells. To assess the cell-autonomous role of Ddb1 in differentiation of CD4^+^ helper T cells, we infected mixed BM chimeric mice with LCMV Armstrong strain after 8 weeks of reconstitution (**Figure 2A**). At day 8 after infection, we found that the frequencies and numbers of total and effector (CD44^+^ CD62L^-^) CD4^+^ T cells derived from Ddb1-TaKO (CD45.1^+^ CD45.2^+^) BM was much lower compared with that derived from WT (CD45.2^+^) BM (**Figures 2B–G**). While we did not observe any difference in the proportion of total T_FH_ cells and GC T_FH_ cells between Ddb1-TaKO and WT effector CD4^+^ T cells, the absolute number of total T_FH_ cells and GC T_FH_ cells was significantly reduced in Ddb1-TaKO CD4^+^ T cells compared with that of WT CD4^+^ T cells, in line with the lower abundance of Ddb1-TaKO effector CD4^+^ T cells (**Figures 2H, I**). Interestingly, the frequency and number of Ddb1-TaKO Th1 cells were also much lower than that of WT Th1 cells (**Figures 2J, K**). These results revealed that the defect of Th1 and T_FH_ differentiation could be caused by impaired generation of effector CD4^+^ T cells in Ddb1-TaKO mice.

**Figure 3:**
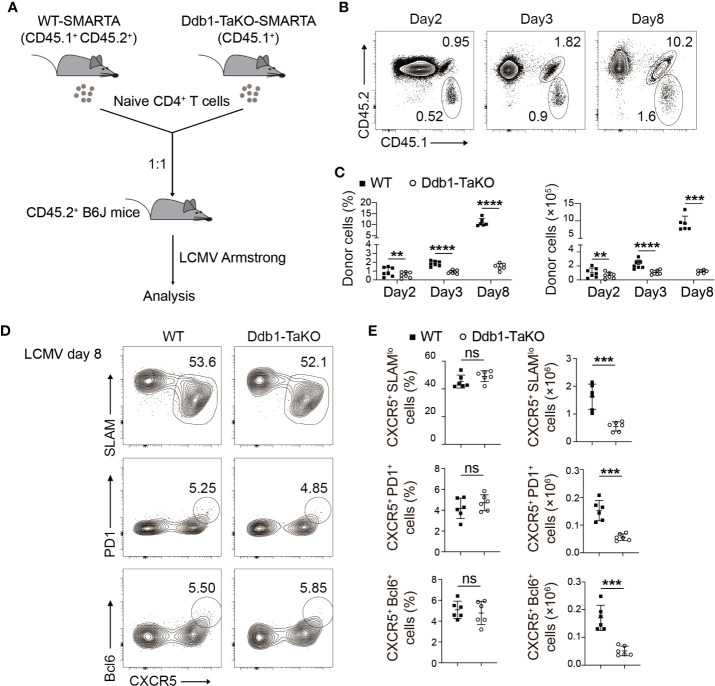
Ddb1 is intrinsically required for expansion of T_FH_ cells but not for their differentiation *per se*. **(A)** Schematic diagram of SMARTA T-cell mix transfer experiments. Donor SMARTA CD4^+^ T cells from Ddb1-TaKO SMARTA mice (CD45.1^+^) and WT SMARTA mice (CD45.1^+^ CD45.2^+^) were mixed in a 1:1 ratio, and cells were adoptively transferred into WT mice (CD45.2^+^), follow by LCMV Armstrong infection. **(B)** Flow cytometry of CD4^+^ T cells in the spleen of mice generated in **(A)**, assessed at days 2, 3, and 8 after LCMV Armstrong infection. Numbers adjacent to outlined areas indicate percentage of donor SMARTA CD4^+^ T cells. **(C)** Frequency (among CD4^+^ T cells) and total cell number of donor SMARTA CD4^+^ T cells in the spleen of mice in **(A)** (n = 6). **(D)** Flow cytometry of donor WT or Ddb1-TaKO SMARTA CD4^+^ T cells in the spleen of mice in **(A)** at day 8 after LCMV Armstrong infection. Numbers adjacent to outlined areas indicate percentage of CXCR5^+^ SLAM^lo^ T_FH_ cells (top) or CXCR5^+^ PD1^+^ GC T_FH_ cells (middle) or CXCR5^+^ Bcl-6^+^ GC T_FH_ cells (bottom). **(E)** Frequency (among donor SMARTA CD4^+^ T cells) and total cell number of T_FH_ cells and GC T_FH_ cells in donor WT or Ddb1-TaKO SMARTA CD4^+^ T cells at day 8 after LCMV Armstrong infection (n = 6). Each symbol represents an individual mouse, small horizontal lines indicate the mean ( ± s.d.). ns, not significant; **P < 0.01; ***P < 0.001; ****P < 0.0001 (Student’s unpaired t-test). Data are representative of three independent experiments (error bars, s.d.).

To test whether Ddb1 was required for clonal expansion and function of effector CD4^+^ T cells during acute viral infection, we crossed Ddb1^fl/fl^; OX40-cre mice with SMARTA TCR transgenic mice (29), which have transgenic expression of a T-cell antigen receptor specific for LCMV GP_61-80_, presented by the major histocompatibility complex (MHC) class II molecule I-A^b^, to generate Ddb1-TaKO SMARTA mice. Naïve CD4^+^ T cells purified from Ddb1-TaKO-SMARTA (CD45.1^+^) and WT-SMARTA (CD45.1^+^ CD45.2^+^) mice were mixed at ratio of 1:1 (**Supplementary Figure S5A**), and then were adoptively transferred into congenic WT recipient mice (CD45.2^+^) followed by LCMV Armstrong infection. At days 2, 3, and 8 after infection, we analyzed frequency and cell number of the donor SMARTA CD4^+^ T cells by flow cytometry (**Figure 3A**). While WT SMARTA CD4^+^ T cells expanded dramatically in response to LCMV Armstrong infection, *Ddb1*-deficient SMARTA CD4^+^ T cells failed to expand in the same hosts (**Figures 3B, C**). In contrast, the activation of WT and Ddb1-TaKO SMARTA CD4^+^ T cells was comparable at both day 3 and day 8 after LCMV Armstrong infection **(** **Supplementary Figures S5B, C**). The frequency and absolute number of early-stage T_FH_ cells of Ddb1-TaKO SMARTA CD4^+^ T cells were similar to that of WT counterparts at day 3 after LCMV Armstrong infection (**Supplementary Figures S5D, E**). Although the frequency of total T_FH_ cells and GC T_FH_ cells was also comparable between WT and Ddb1-TaKO SMARTA CD4^+^ T cells at day 8 after LCMV Armstrong infection, the absolute numbers of total T_FH_ cells and GC T_FH_ cells were much lower in Ddb1-TaKO SMARTA CD4^+^ T cells than that of WT counterparts, due to severe defect of expansion in Ddb1-TaKO SMARTA CD4^+^ T cells (**Figures 3D, E**). Collectively, these results demonstrated that Ddb1 was essential for T_FH_ and Th1 cell expansion in a cell-intrinsic manner but not for T_FH_ differentiation per se.

### Deletion of *Ddb1* Leads to Cell Cycle Arrest and Increases Cell Death

**Figure 4:**
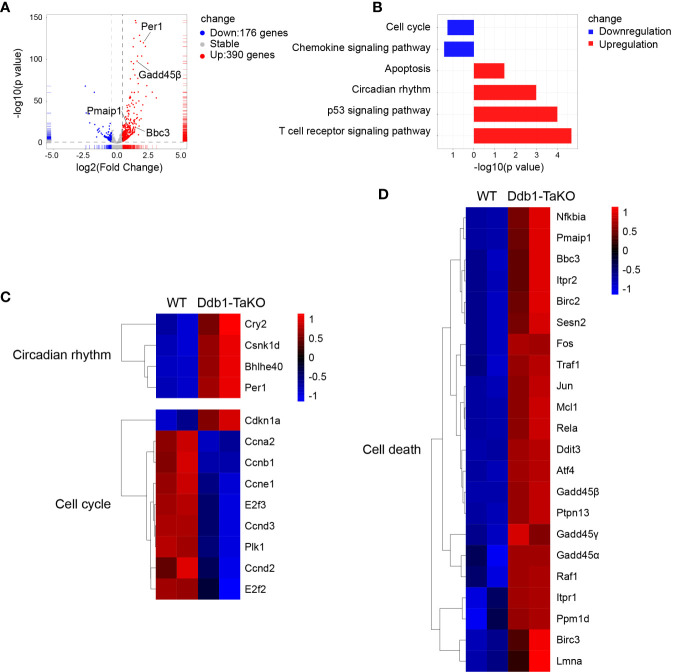
Cell cycle and cell death associated genes are enriched in *Ddb1*-deficient T cells. **(A)** Volcano plots of the differentially expressed genes (DEGs) in Ddb1-TaKO SMARTA CD4^+^ T cells relative to the expression in WT SMARTA CD4^+^ T cells, by RNA-seq analysis of Ddb1-TaKO SMARTA CD4^+^ T cells and WT SMARTA CD4^+^ T cells isolated from B6 mice 5d after transfer of SMARTA cells and infection of hosts with LCMV Armstrong. **(B)** The KEGG pathway enrichment analysis of DEGs from mice in **(A)**. **(C, D)** Heat map of selected genes of DEGs from mice in **(A)**.

To elucidate which signaling pathways were affected by Ddb1 deficiency, we sorted Ddb1-TaKO and WT SMARAT cells in mixed transfer experiments at day 5 after LCMV Armstrong infection, and performed RNA-sequencing (RNA-seq) analysis of these cells. We found that 176 genes were downregulated and 390 genes were upregulated in Ddb1-deficient SMARTA CD4^+^ T cells relative to those in WT SMARTA CD4^+^ T cell (**Figure 4A**). Kyoto Encyclopedia of Genes and Genomes (KEGG) analysis uncovered that Ddb1-TaKO SMARTA CD4^+^ T cells downregulated pathways related to cell cycle progression and upregulated pathways associated with cell death and p53 signaling (**Figure 4B**). In detail, the expression of genes in cell cycle including Plk1 and Cyclin was decreased, and that of genes in cell death and p53 signaling pathways including Gadd45 family, P21, Bbc3, and Pmaip1 was increased in *Ddb1*-deficient CD4^+^ T cells compared to WT CD4^+^ T cells (**Figures 4C, D**). All these results implied that Ddb1 might have distinct functions in proliferation and cell death of activated CD4^+^ T cells.

**Figure 5:**
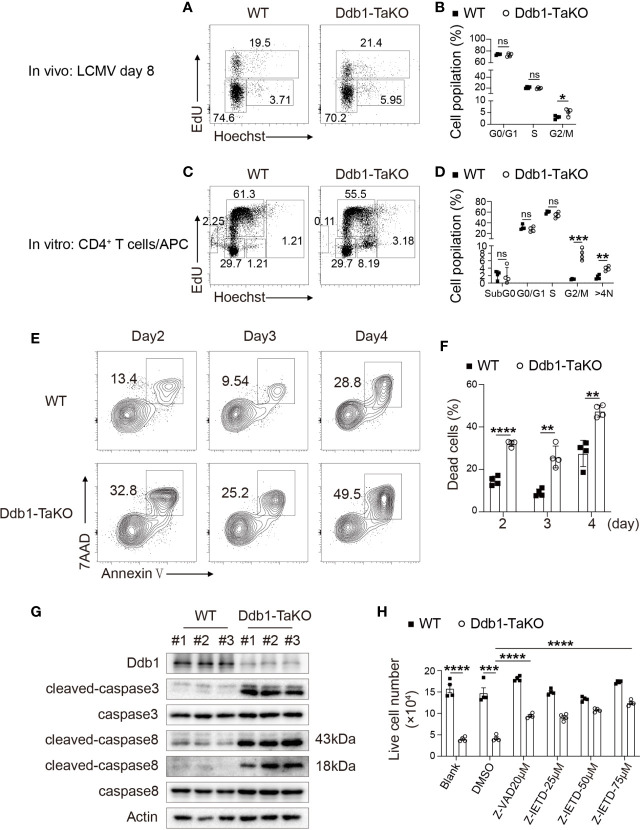
Deletion of Ddb1 leads to cell cycle arrest and caspase-dependent cell death. **(A)** Cell cycle analysis of activated CD4^+^ T cells by EdU incorporation assay in the spleen of Ddb1-TaKO and WT mice at day 8 after LCMV Armstrong infection. Percentage of each phase in cell cycle is shown on the graph. **(B)** Frequency (among activated CD4^+^ T cells) of each phase in cell cycle in the spleen of mice as in **(A)** (n = 4). **(C)** Naïve CD4^+^ T cells purified from Ddb1-TaKO and WT mice were co-cultured with APCs in the presence of 1 μg/ml anti-CD3 and anti-CD28. Cell cycle analysis of CD4^+^ T cells by EdU incorporation assay at day 3. Percentage of each phase is shown on the graph. **(D)** Frequency of each phase in cell cycle as in **(C)**. **(E)** Flow cytometry of *in vitro* activated CD4^+^ T cells in co-cultured system for indicated days as in **(C)** Numbers adjacent to outlined areas indicate percentage of Annexin V^+^ 7AAD^+^ apoptotic CD4^+^ T cells. **(F)** Frequency of apoptotic (Annexin V^+^ 7AAD^+^) CD4^+^ T cells in **(E)**. **(G)** Immunoblot analysis of lysates of *in vitro* activated Ddb1-TaKO and WT CD4^+^ T cells in co-cultured system for 3 days as in **(C)**, probed with anti-caspase antibodies. **(H)** Live cell number of *in vitro* activated Ddb1-TaKO and WT CD4^+^ T cells in co-cultured system in the presence or absence of caspase inhibitors for 3 days as in **(C)** Each symbol represents an individual mouse or individual well of cells; small horizontal lines indicate the mean ( ± s.d.). ns, not significant; *P < 0.05; **P < 0.01; ***P < 0.001; ****P < 0.0001 (Student’s unpaired t-test). Data are representative of three independent experiments (error bars, s.d.).

Next, we examined whether impaired CD4^+^ T-cell expansion was caused by proliferation defects. The hallmark for cell cycle entry is DNA synthesis and subsequent cell division. To investigate the role of Ddb1 in cell cycle progression, EdU incorporation assay was performed in Ddb1-TaKO mice and WT mice at day 8 after LCMV Armstrong infection. According to Hoechst and EdU staining, we found that *Ddb1* deficiency led to cell cycle arrest at G2/M phase in activated CD4^+^ T cells, while G1 and S phase remained unaltered (**Figures 5A, B**). To further demonstrate whether *Ddb1* deletion causes G2/M arrest, we also analyzed the cell cycle progression in a co-culture system and found that naive CD4^+^ T cells isolated from Ddb1-TaKO mice co-cultured with antigen presenting cells (APCs) also displayed G2/M arrest (**Figures 5C, D**). Furthermore, Ddb1 deletion increased the percentage of cells with G2/M arrest and of the cells with DNA content more than 4n (**Figures 5C, D**). These results suggested that Ddb1 regulated cell cycle progression by controlling G2/M transition and prevented activated CD4^+^ T cells from allopolyploid formation.

To test whether impaired CD4^+^ T-cell expansion in Ddb1-TaKO cells was due to increase of cell death, we examined apoptosis status in the co-culture system. First, we quantified the lived cell number of Ddb1-TaKO and WT cells at days 0–6 after co-culture with APCs. As expected, the cell number of Ddb1-TaKO activated CD4^+^ T cells was much lower compared with WT activated CD4^+^ T cells at days 2–6 (**Supplementary Figure S6A**). To determine whether Ddb1 deficiency in activated CD4^+^ T cells enhance T-cell apoptosis, cells were stained with Annexin V/7AAD followed by flow cytometry analysis. Compared with WT activated CD4^+^ T cells, there was a significant increase in frequency and number of apoptosis cells in Ddb1-TaKO activated CD4^+^ T cells (**Figures 5E, F**). Furthermore, *Ddb1* deficiency resulted in much more accumulation of cleaved caspase-3 and caspase-8 (**Figure 5G**). Besides that, pan caspase inhibitor Z-VAD-FMK, and caspase-8 inhibitor Z-IETD-FMK, at least partially blocked cell death in activated Ddb1-TaKO CD4^+^ T cells (**Figure 5H**), whereas ferroptosis inhibitor Ferrostatin-1 and necroptosis inhibitor GSK872/NEC-1/NEC1s did not rectify cell death in activated Ddb1-TaKO CD4^+^ T cells (**Supplementary Figure S6B**). These data suggested that the elevated cell death of activated CD4^+^ T cells in Ddb1-TaKO mice was mainly caused by caspase-dependent apoptosis. Collectively, these results indicated that Ddb1 was vital in CD4^+^ T-cell expansion by controlling cell cycle progression and cell death.

### Defective T_FH_ Generation Independent of p53 Accumulation

We further investigated whether *Ddb1* deletion affected the expression of critical regulators for cell cycle progression and apoptosis. As p53 has been shown to be critical for cell cycle progression and apoptosis, inactivation of Cul4-Ddb1-DCAFs stabilized p53 (30, 31). Furthermore, it has been reported that deletion of Dcaf1 or Dcaf2 in mature T cells caused severe defect in peripheral T-cell homeostasis and antigen-induced expansion (32, 33). To delineate whether the regulation of T_FH_ cell differentiation by Ddb1 was dependent on the Cul4-Ddb1-DCAFs complexes, we crossed Cul4a^fl/fl^ Cul4b^fl/fl^ mice with OX40-cre mice to generate Cul4a^fl/fl^ Cul4b^fl/fl^; OX40-cre (Cul4a/b-DTaKO) mice. At day 8 after LCMV Armstrong infection, Cul4a/b-DTaKO mice with deletion of both *Cul4a* and *Cul4b* exhibited similar defective T_FH_ cell and B-cell response to Ddb1-TaKO mice, indicating that an E3 ligase function of the Cul4-Ddb1-DCAFs complexes was crucial for T_FH_ cell generation (**Supplementary Figures S7A–D**).

**Figure 6:**
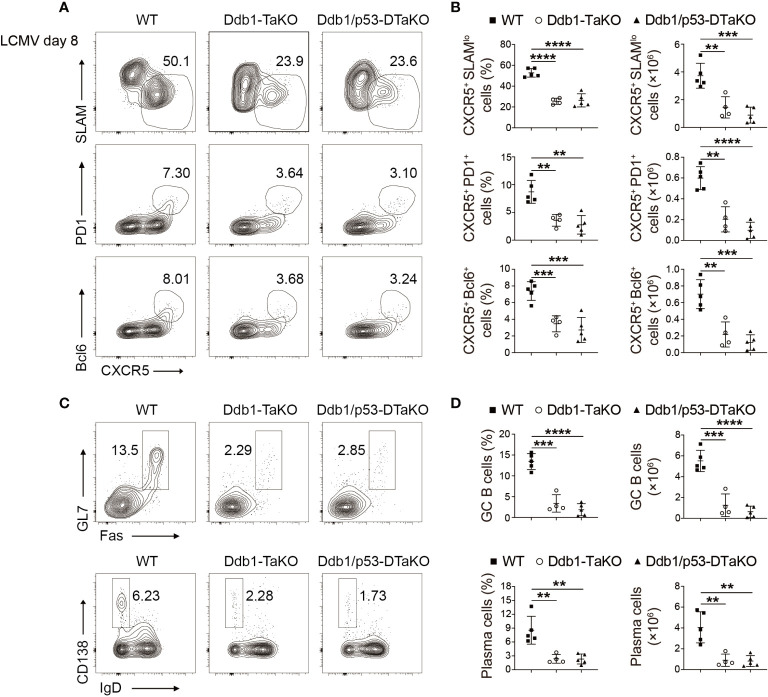
p53 ablation does not rectify the defective T_FH_ generation in Ddb1-TaKO mice. **(A)** Flow cytometry of activated (CD44^hi^) CD4^+^ T cells from WT, Ddb1-TaKO, and Ddb1^fl/fl^ Trp53^fl/fl^ OX40-cre (Ddb1/p53-DTaKO) mice at day 8 day after LCMV Armstrong infection. Numbers adjacent to outlined areas indicate percentage of CXCR5^+^ SLAM^lo^ polyclonal T_FH_ cells (top row) or CXCR5^+^ PD1^+^ GC T_FH_ cells (middle row) or CXCR5^+^ Bcl-6^+^ GC T_FH_ cells (bottom row). **(B)** Frequency and total number of T_FH_ cells and GC T_FH_ cells in the spleen of mice as in **(A)** (n = 5). **(C)** Flow cytometry of total B220^+^ B cells from mice as in **(A)** Number adjacent to outlined areas indicate percentage of GC B and plasma cells. **(D)** Frequency and total number of GC B and plasma cells in the spleen of mice as in **(A)** (n = 5). Each symbol represents an individual mouse, small horizontal lines indicate the mean ( ± s.d.). **P < 0.01; ***P < 0.001; ****P < 0.0001 (Student’s unpaired t-test). Data are representative of three independent experiments (error bars, s.d.).

Next, we tested if *Ddb1* deletion affected the expression of p53 and whether p53 contributed the phenotype of Ddb1-TaKO mice. First, we examined p53 expression in Ddb1 deficient activated CD4^+^ T cells in co-culture system with APCs by Western blot. Consistent with previous report (30, 31), the expression of p53 protein was also higher in *Ddb1*-deficient than in WT activated CD4^+^ T cells (**Supplementary Figure S8**). To test whether p53 is involved in Ddb1 regulation of cell cycle progression and apoptosis, we crossed mice with *Trp53* ^fl/fl^ to *Ddb1* ^fl/fl^; OX40-cre to generate mice deficient in both *Ddb1* and *Trp53* (*Ddb1* ^fl/fl^ *Trp53* ^fl/fl^ OX40-cre, Ddb1/p53-DTaKO) mice. We infected the progeny with LCMV Armstrong and analyzed their T-cell and B-cell responses at day 8 after infection. The differentiation of T_FH_ and GC T_FH_ as well as GC B and plasma cells in Ddb1/p53-DTaKO was substantially impaired compared with that in WT mice, and was not greater than that in Ddb1-TaKO mice, which suggested that the defects in T_FH_ differentiation and GC response could not be restored by deletion of p53 (**Figures 6A–D**). Together, these findings suggested that Ddb1 regulated cell cycle progression and apoptosis of effector CD4^+^ T cells at least partially in a p53-independent pathway.

### *Ddb1* Deficiency Causes Aberrant DNA Damage Responses

**Figure 7:**
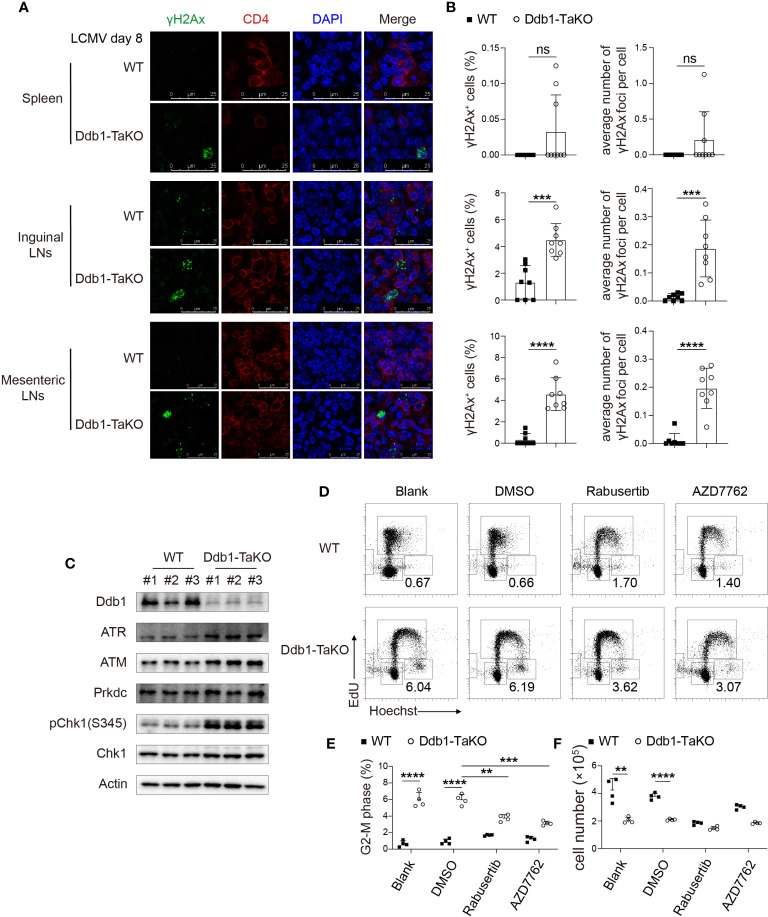
Ddb1 deficiency causes accumulation of DNA damage and hyper-activation of ATM/ATR-Chk1 pathway. **(A)** Immunofluorescence of γH2Ax frozen sections in the spleen, inguinal LNs, and mesenteric LNs from WT and Ddb1-TaKO mice at day 8 after LCMV Armstrong infection. **(B)** Frequency of γH2Ax^+^ activated CD4^+^ T cells and the number of γH2Ax foci per cell were quantitated. **(C)** Immunoblot analysis of lysates of Ddb1-TaKO and WT activated CD4^+^ T cells at day 3 in **Figure 5C**, probed with antibodies to Chk1 signaling related proteins. **(D)** Cell cycle analysis of CD4^+^ T cells treated with Chk1 inhibitors by EdU incorporation assay at day 3 in **Figure 5C**. Numbers adjacent to outlined areas indicate percentage of CD4^+^ T cells at G2-M phase. **(E)** Frequency of CD4^+^ T cells at G2-M phase in **(D)**. **(F)** Total number of activated CD4^+^ T cells in **(D)** Each symbol represents an individual mouse or individual well of cells, small horizontal lines indicate the mean ( ± s.d.). ns, not significant; **P < 0.01; ***P < 0.001; ****P < 0.0001 (Student’s unpaired t-test). Data are representative of three independent experiments (error bars, s.d.).

Ddb1 functions in nucleotide-excision repair (NER) and binds to DNA following UV damage (34). Defective activity of UV-DDB causes defective DNA repair in patients with Xeroderma pigmentosum complementation group E (XP-E), an autosomal recessive disorder characterized by photosensitivity and early onset of carcinomas (35). To investigate whether *Ddb1* deletion in activated CD4^+^ T cells resulted in genomic stress with DNA damage accumulation, we sacrificed WT and Ddb1-TaKO mice after LCMV Armstrong infection and then stained CD4^+^ T-cell with antibodies against histone H2AX phosphorylated at serine 139 (γH2Ax) to assess DNA damage. We found that γH2Ax accumulation was increased in activated CD4^+^ T cells from the lymphoid organs of Ddb1-TaKO mice compared with that of WT mice (**Supplementary Figures S9A, B**). Furthermore, we confirmed γH2Ax accumulation by immunofluorescence and found that there were much more γH2Ax specks in inguinal and mesenteric lymph nodes and slightly more in spleens of Ddb1-TaKO mice than in that of WT mice (**Figures 7A, B**). Together these data suggested that loss of Ddb1 in activated CD4^+^ T cells led to accumulation of DNA damage during acute viral infection.

The ATM/ATR-Chk1 signaling pathway is essential for G2/M arrest following DNA damage (36, 37). Therefore, we determined whether these signaling pathways were activated in Ddb1-TaKO mice. Importantly, Chk1 phosphorylation and protein levels of ATM and ATR were dramatically enhanced in activated Ddb1-TaKO CD4^+^ T cells compared with activated WT CD4^+^ T cells (**Figure 7C**), suggesting that hyper activation of ATM/ATR-Chk1 pathway may contribute to cell cycle arrest in Ddb1-TaKO mice. To further evaluate the effects of Chk1 on G2/M arrest in activated CD4^+^ T cells, we treated Ddb1-TaKO and WT CD4^+^ T cells with Rabusertib and AZD7762 (Chk1 inhibitors) in a co-culture system. Chk1 inhibitors partially reversed G2-M arrest in Ddb1-TaKO cells (**Figures 7D, E**). However, the numbers of Ddb1-TaKO live cells were not improved by Chk1 inhibitors (**Figure 7F**), indicating that DNA damage caused by *Ddb1* deficiency, triggered both cell death and ATM/ATR-Chk1-mediated cell cycle arrest. Thus, these results also confirmed that ATM/ATR-Chk1 signaling pathway played a key role in controlling cell cycle at G2-M phase.

## Discussion

Ddb1, an DNA damage binding protein, plays an important regulatory role in diverse cellular functions. A previous study showed that deficiency of *Ddb1* gene in mice leads to early embryonic lethality, and deletion of Ddb1 in the brain results in elimination of neuronal progenitor cells, hemorrhage in brain, and neonatal lethality (25). Another study found that *Ddb1* deletion impairs function of hematopoietic stem cells in the bone marrow and fetal liver (38). However, the function of Ddb1 in immune cells is largely unknown, especially in effector CD4^+^ T cells. Here, we showed that deletion of Ddb1 in effector CD4^+^ T cells led to impaired T_FH_ and Th1 cell generation due to defect of T-cell expansion upon acute viral infection. Although Treg cell-specific deletion of Ddb1 led to impaired stability and function of Treg cells and development of early onset fatal autoimmune disease (manuscript in preparation), our data with experiments with both mixed bone marrow chimera and mixed SMARTA transfer suggested that Ddb1 was required for T_FH_ and Th1 cell generation by controlling CD4^+^ T-cell expansion in a cell-intrinsic manner.

Our findings demonstrated that deletion of Ddb1 in effector CD4^+^ T cells led to accumulation of DNA damage, proliferation defect, and increase of cell death. Previous study has identified p53 as a substrate of Cul4-Ddb1-DCAFs E3 ligase complex, and brain-specific deletion of Ddb1 leads to selective elimination of proliferating neuronal progenitor cells by apoptosis and that partially rescued by removal of p53 (25). Consistent with previous studies, Ddb1-TaKO mice displayed accumulation of p53 in effector CD4^+^ T cells. However, p53 deletion did not rescue the phenotype of Ddb1-TaKO mice, indicating that p53-independent pathway controls the expansion of effector CD4^+^ T cells.

One possible pathway is accumulation of unrepaired DNA damage and genomic stress during clonal expansion of effector CD4^+^ T cells. In general, DNA damage can be repaired by NER pathway quickly without affecting cell cycle progression. Therefore, mutations in NER components such as Ddb1 deletion cause aberrant accumulation of DNA damage that lead to activation of the ATM/ATR-Chk1 and p53 pathway and consequently promotes cell cycle arrest and cell death. These findings are consistent with a very recent report that ablation of Cul4b in mature T cells resulted in impaired survival and proliferation accompanied by accumulation of DNA damage upon T-cell stimulation (39). This recent study further demonstrated that Cul4-Ddb1-DCAF1 E3 ligase complex associated with a DNA damage repair complex including SMC1A and promoted phosphorylation of Smc1a to aid in DNA damage repair (39).

As Ddb1 is crucial for activity of Cul4-Ddb1-DCAFs E3 ligase complex, deletion of Ddb1 in effector CD4^+^ T cells could result in augmented protein levels of substrates involved in regulation of cell cycle, such as Cdt1 (18, 19), p21 (20, 21), and Cyclin D (40–42). Hence, aberrant accumulation of these substrates may also cause cell cycle arrest and cell death of effector CD4^+^ T cells. Ultimately, we speculate that increased DNA damage and abnormal accumulation of Ddb1-mediated degraded protein substrates, including p53, Cdt1, p27, and Cyclin D, could account for the development and function defects of T_FH_ and Th1 cells in Ddb1-TaKO mice.

Overall, our findings have established a critical function for Ddb1 as positive regulator in T-cell expansion during CD4^+^ helper T-cell differentiation. We have further shown that Ddb1 promotes T-cell expansion by preventing DNA damage-induced cell cycle arrest and cell death. Thus, understanding how Ddb1 regulates such differentiation process may shed light on rational vaccine design for infectious disease.

## Data Availability Statement

RNA-seq datasets have been deposited in the Gene Expression Omnibus (GEO) (https://www.ncbi.nlm.nih.gov/geo/query/acc.cgi) with accession numbers GSE176437.

## Ethics Statement

The animal study was reviewed and approved by the Institutional Animal Care and Use Committee of Xiamen University.

## Author Contributions

LY and WC designed and executed the experiments, analyzed the data, and wrote the manuscript. QZ analyzed RNA-seq data under the supervision of QL. LL, YX, SF, TX, and ML performed the experiments. YH and TZ provided expertise and materials. W-HL helped with the experimental design and data analysis and wrote the paper. NX designed the experiments, analyzed the data, and wrote the manuscript with input from all authors. All authors contributed to the article and approved the submitted version.

## Funding

This study was supported by the National Natural Science Foundation of China (31770955 and 31570883 to NX, and 31570882 and 31770950 to WL), 1000 Young Talents Program of China (NX), and the Fundamental Research Funds for the Central Universities of China-Xiamen University (20720150065 to NX).

## Conflict of Interest

The authors declare that the research was conducted in the absence of any commercial or financial relationships that could be construed as a potential conflict of interest.

## Publisher’s Note

All claims expressed in this article are solely those of the authors and do not necessarily represent those of their affiliated organizations, or those of the publisher, the editors and the reviewers. Any product that may be evaluated in this article, or claim that may be made by its manufacturer, is not guaranteed or endorsed by the publisher.

## Supplementary Material

Supplementary Figure 1 The expression of Ddb1 in immune cells and genetic ablation of Ddb1 in activated CD4^+^ T cells. **(A)** The mRNA levels of Ddb1 in indicated immune cells (Database: Gene Atlas MOE430, gcrma from BioGPS). **(B)** Immunoblot analysis of Ddb1 in indicated immune cells. **(C)** Representative genotyping PCR analysis of tail DNA from WT, *Ddb1* ^fl/+^, *Ddb1* ^fl/fl^ mice displayed on a 2% agarose gel. **(D)** Immunoblot analysis of Ddb1 in naïve CD4^+^ T cells purified from WT and Ddb1-TaKO mice and stimulated with 3μg/ml anti-mouse CD3 and anti-mouse CD28 for indicated times. Data are representative of three independent experiments (error bars, s.d.).

Supplementary Figure 2 Hyperactivation of peripheral T cells in Ddb1-TaKO mice. **(A)** Flow cytometry of thymocytes from WT and Ddb1-TaKO mice at 6 weeks of age. **(B)** Frequency (among total thymocytes) and total number of DN, DP, CD4^+^, and CD8^+^ SP populations from **(A)** (n = 4). **(C)** Flow cytometry of splenocytes from WT and Ddb1-TaKO mice. Numbers adjacent to outlined areas indicate percentage of CD4^+^ and CD8^+^ T cells. **(D)** Frequency (among total splenocytes) and total number of CD4^+^ T cells and CD8^+^ T cells from **c** (n = 4). **(E)** Flow cytometry of CD4^+^ T (top row) and CD8^+^ T (bottom row) cells in the spleen from WT and Ddb1-TaKO mice. Numbers adjacent to outlined areas indicate percentage of effector (CD44^hi^ CD62L^lo^) T cells. **(F)** Frequency (among CD4^+^ or CD8^+^ T cells) and total number of effector (CD44^hi^ CD62L^lo^) T cells from **e** (n = 4). Each symbol represents an individual mouse; small horizontal lines indicate the mean ( ± s.d.). ns, not significant; *P < 0.05; **P < 0.01; ***P < 0.001; ****P < 0.0001 (Student’s unpaired t-test.). Data are representative of three independent experiments (error bars, s.d.).

Supplementary Figure 3 Ddb1 is required for peripheral T-cell homeostasis. Flow cytometry of thymocyte development and peripheral T cells in mixed BM chimeric mice. **(A)** Flow cytometry of thymocytes in chimeras. Numbers adjacent to outlined areas indicate percentage of WT (CD45.1^+^ CD45.2^+^) or Ddb1-TaKO (CD45.2^+^) donor cells. **(B)** Frequency (among thymocytes) and total cell number of WT or Ddb1-TaKO donor cells in thymus of mice in **a** (n = 5). **(C)** Flow cytometry of splenocytes in chimeras. Numbers adjacent to outlined areas indicate percentage of WT or Ddb1-TaKO donor cells. **(D)** Frequency (among splenocytes) and total cell number of WT or Ddb1-TaKO donor cells in spleen of mice in **a** (n = 5). **(E)** Flow cytometry of WT or Ddb1-TaKO donor thymocytes in chimeras. **(F)** Frequency (among WT or Ddb1-TaKO donor thymocytes) and total number of DN, DP, CD4^+^, and CD8^+^ SP populations (n = 5). **(G)** Flow cytometry of WT or Ddb1-TaKO donor cells in the spleen of chimeras. Number adjacent to outlined areas indicate percentage of CD4^+^ or CD8^+^ T cells. **(H)** Frequency and total number of CD4^+^ T or CD8^+^ T cells in WT or Ddb1-TaKO donor splenocytes (n = 5). **(I)** Flow cytometry of CD4^+^ T and CD8^+^ T cells in WT or Ddb1-TaKO donor cells. Numbers adjacent to outlined areas indicate percentage of effector (CD44^hi^ CD62L^lo^) CD4^+^ T or CD8^+^ T cells. **(J)** Frequency and total number of effector (CD44^hi^ CD62L^lo^) CD4^+^ T or CD8^+^ T cells in WT or Ddb1-TaKO donor cells (n = 5). Each symbol represents an individual mouse; small horizontal lines indicate the mean ( ± s.d.). ns, not significant; *P < 0.05; **P < 0.01; ***P < 0.001; ****P < 0.0001 (Student’s unpaired t-test.). Data are representative of three independent experiments (error bars, s.d.).

Supplementary Figure 4 Th1 cell responses in Ddb1-TaKO mice after LCMV infection. **(A)** Flow cytometry of splenocytes from mice in **Figure 1C**. Number adjacent to outlined areas indicate percentage of CD4^+^ T cells. **(B)** Frequency and total number of CD4^+^ T cells in **(A)** (n = 4). **(C)** Flow cytometry of CD4^+^ T cells in the spleen from WT and Ddb1-TaKO mice. Numbers adjacent to outlined areas indicate percentage of effector (CD44^hi^ CD62L^lo^) CD4^+^ T cells. **(D)** Frequency (among CD4^+^ T cells) and total number of effector (CD44^hi^ CD62L^lo^) T cells from **(C)** (n = 4). **(E)** Flow cytometry of splenic CD4^+^ T cells from mice in **Figure 1C**. Number adjacent to outlined areas indicate percentage of IFNγ^+^ CD4^+^ T cells. **(F)** Frequency (among CD4^+^ T cells) and total number of IFNγ^+^ CD4^+^ T cells in **(E)** (n = 4). Each symbol represents an individual mouse, small horizontal lines indicate the mean ( ± s.d.). *P < 0.05; **P < 0.01; ***P < 0.001 (Student’s unpaired t-test). Data are representative of three independent experiments (error bars, s.d.).

Supplementary Figure 5 Normal activation and T_FH_ differentiation in *Ddb1*-deficient SMARTA CD4^+^ T cells. **(A)** Flow cytometry of indicated donor naïve CD4^+^ T cells before adoptive transfer in **Figure 3A**. **(B)** Flow cytometry of donor Ddb1-TaKO and WT SMARTA CD4^+^ T cells in the spleen of mice in **Figure 3A** at days 3 and 8 after LCMV Armstrong infection. Numbers adjacent to outlined areas indicate percentage of CD44^+^ CD4^+^ T cells. **(C)** Frequency (among SMARTA CD4^+^ T cells) and total cell number of CD44^+^ CD4^+^ T cells in **(B)** (n = 6). **(D)** Flow cytometry of donor Ddb1-TaKO and WT SMARTA CD4^+^ T cells in the spleen of mice in **Figure 3A** at day 3 after LCMV Armstrong infection. Numbers adjacent to outlined areas indicate percentage of early-stage CXCR5^+^ SLAM^lo^ T_FH_ cells (top row) or CXCR5^+^ PD1^+^ T_FH_ cells (middle row) or CXCR5^+^ Bcl-6^+^ T_FH_ cells (bottom row). **(E)** Frequency (among SMARTA CD4^+^ T cells) and total cell number of early-stage T_FH_ cells in **(D)** (n = 6). Each symbol represents an individual mouse; small horizontal lines indicate the mean ( ± s.d.). ns, not significant; **P < 0.01; ****P < 0.0001 (Student’s unpaired t-test.). Data are representative of three independent experiments (error bars, s.d.).

Supplementary Figure 6 Inhibitors of ferroptosis and necroptosis do not improve survival of *Ddb1*-deficient activated CD4^+^ T cells. **(A)** Live cell number of Ddb1-TaKO and WT activated CD4^+^ T cells at days 0–6 in **Figure 5C**. **(B)** Live cell number of Ddb1-TaKO and WT activated CD4^+^ T cells treated with 5 μM Ferrostatin-1, 2 μM GSK872, 50 μM NEC1, and 50 μM NEC1s at day 3 in **Figure 5C**. Each symbol represents an individual well of cells; small horizontal lines indicate the mean ( ± s.d.). ***P < 0.001; ****P < 0.0001 (Student’s unpaired t-test.). Data are representative of three independent experiments (error bars, s.d.).

Supplementary Figure 7 The Cul4-Ddb1 E3 ligase complex is required for T_FH_ cell generation. Flow cytometry of activated (CD44^hi^) CD4^+^ T cells from WT, Ddb1-TaKO, and Cul4a^fl/fl^ Cul4b^fl/fl^; OX40-cre (Cul4a/b-DTaKO) mice at day 8 after LCMV Armstrong infection. **(A)** Numbers adjacent to outlined areas indicate percentage of CXCR5^+^ SLAM^lo^ polyclonal T_FH_ cells (top row) or CXCR5^+^ PD1^+^ GC T_FH_ cells (middle row) or CXCR5^+^ Bcl-6^+^ GC T_FH_ cells (bottom row). **(B)** Frequency and total number of T_FH_ and GC T_FH_ cells in the spleen of mice as in **(A)** (n = 3). **(C)** Flow cytometry of total B220^+^ B cells from mice as in **(A)**. Number adjacent to outlined areas indicate percentage of GC B and plasma cells. **(D)** Frequency and total number of GC B and plasma cells in the spleen of mice as in **(A)** (n = 3). Each symbol represents an individual mouse; small horizontal lines indicate the mean ( ± s.d.). *P < 0.05; **P < 0.01; ***P < 0.001; ****P < 0.0001 (Student’s unpaired t-test.). Data are representative of three independent experiments (error bars, s.d.).

Supplementary Figure 8 p53 accumulation in *Ddb1*-deficient activated CD4^+^ T cells. Immunoblot analysis of Ddb1, p53 in Ddb1-TaKO, and WT activated CD4^+^ T cells at day 3 in **Figure 5C**. Data are representative of three independent experiments.

Supplementary Figure 9 Ddb1 deletion results in the accumulation of DNA damage. **(A)** Flow cytometry of activated (CD44^hi^) CD4^+^ T cells from WT and Ddb1-TaKO mice 8 days after infection with LCMV Armstrong strain. Numbers adjacent to outlined areas indicate γH2Ax^+^ activated CD4^+^ T cells. **(B)** Frequency (among activated (CD44^hi^) CD4^+^ T cells) and cell number of γH2Ax^+^ activated CD4^+^ T cells in the spleen, inguinal LNs, and mesenteric LNs in **(A)** (n = 3). Each symbol represents an individual mouse; small horizontal lines indicate the mean ( ± s.d.). ns, not significant; *P < 0.05; **P < 0.01; ***P < 0.001 (Student’s unpaired t-test.). Data are representative of three independent experiments (error bars, s.d.).
