# Supplementary figures and images for: Ddb1 Is Essential for the Expansion of CD4+ Helper T Cells by Regulating Cell Cycle Progression and Cell Death
Source: Front Immunol. 2021 Aug 30;12:722273. doi: 10.3389/fimmu.2021.722273 (PMC8435776; doi:10.3389/fimmu.2021.722273)

Fig. S1

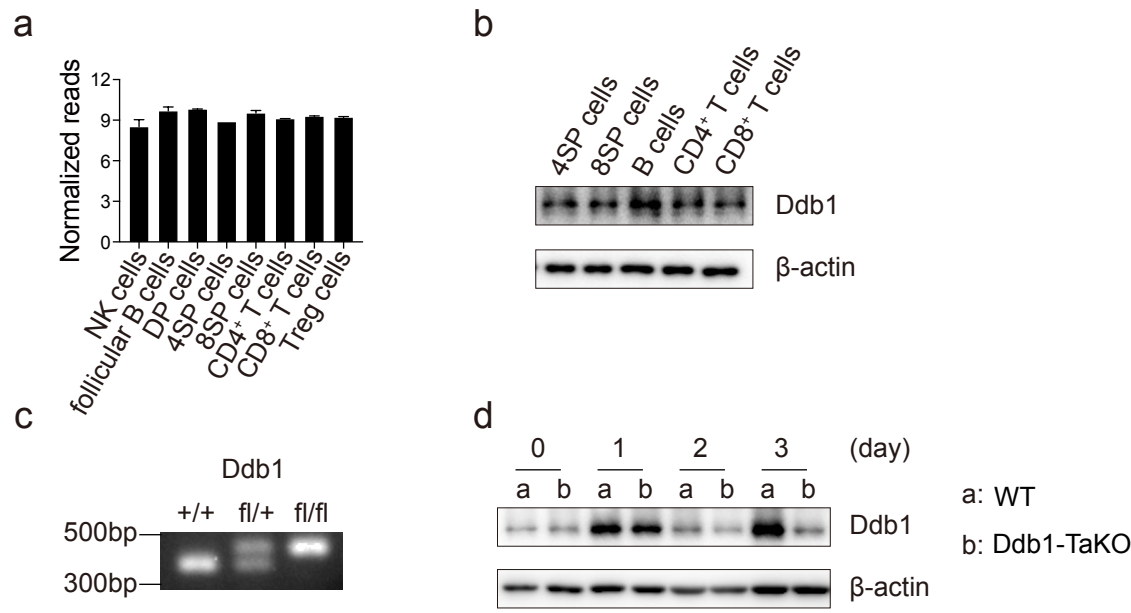

Fig. S2

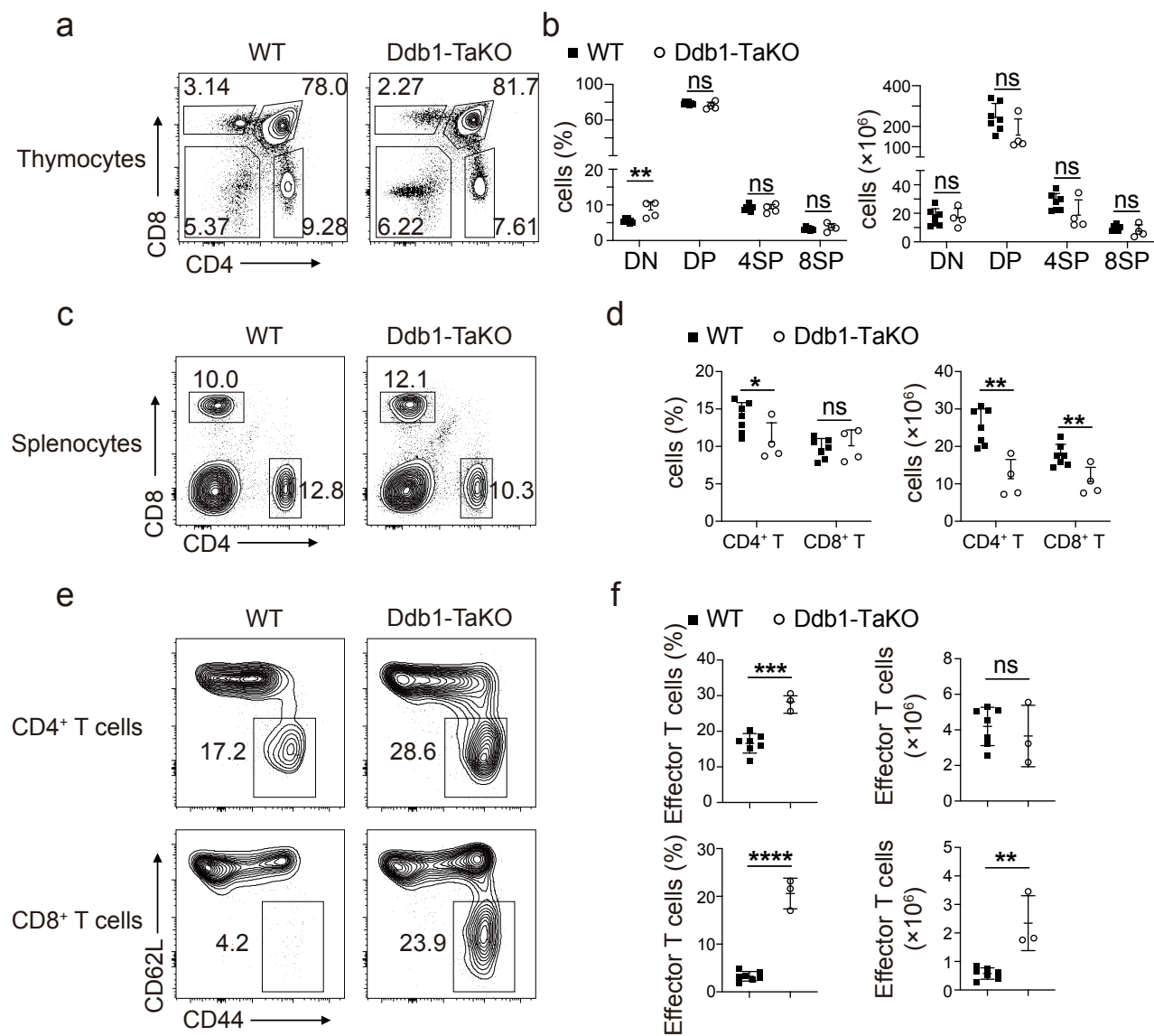

Fig. S3

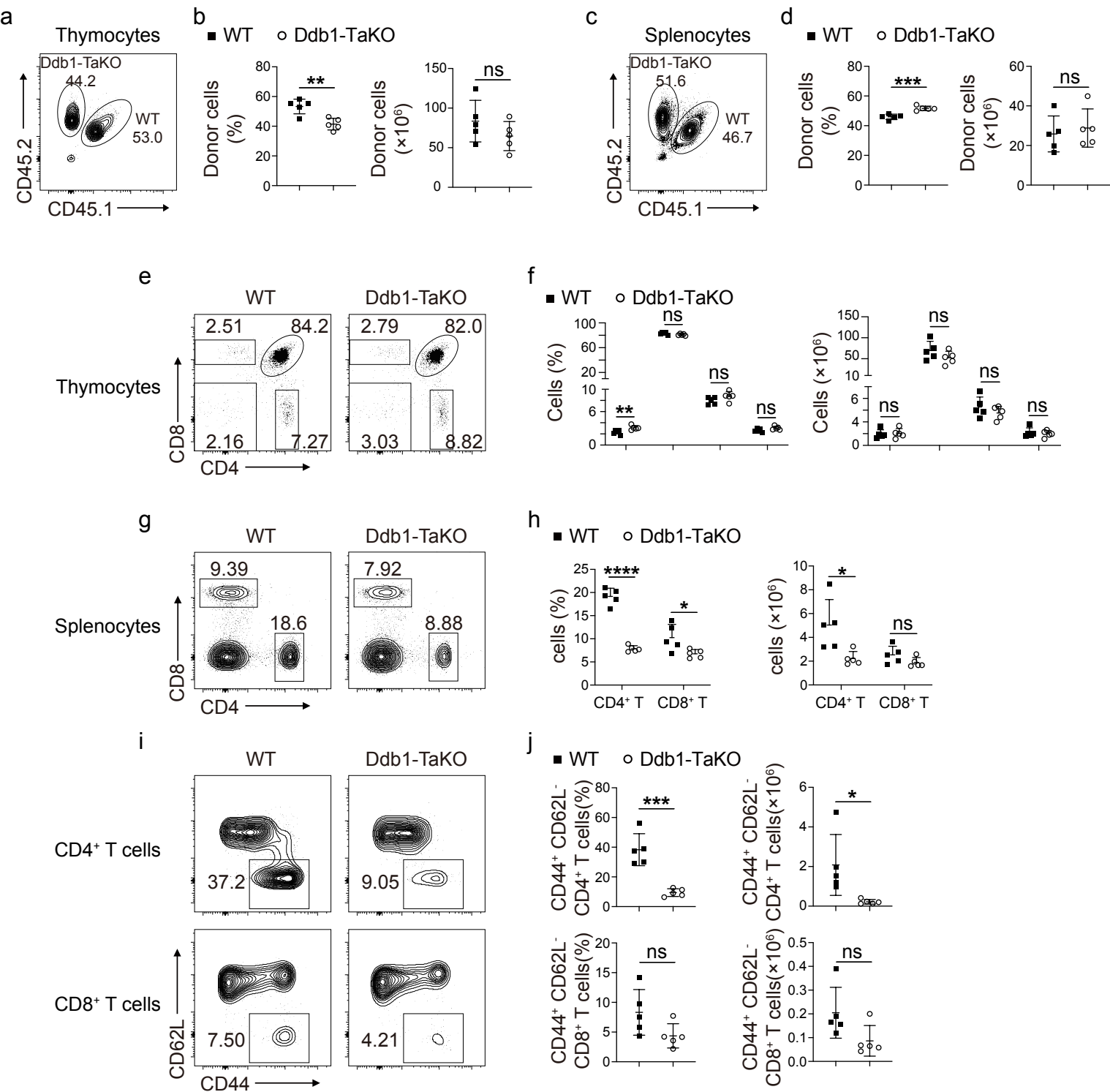

Fig. S4

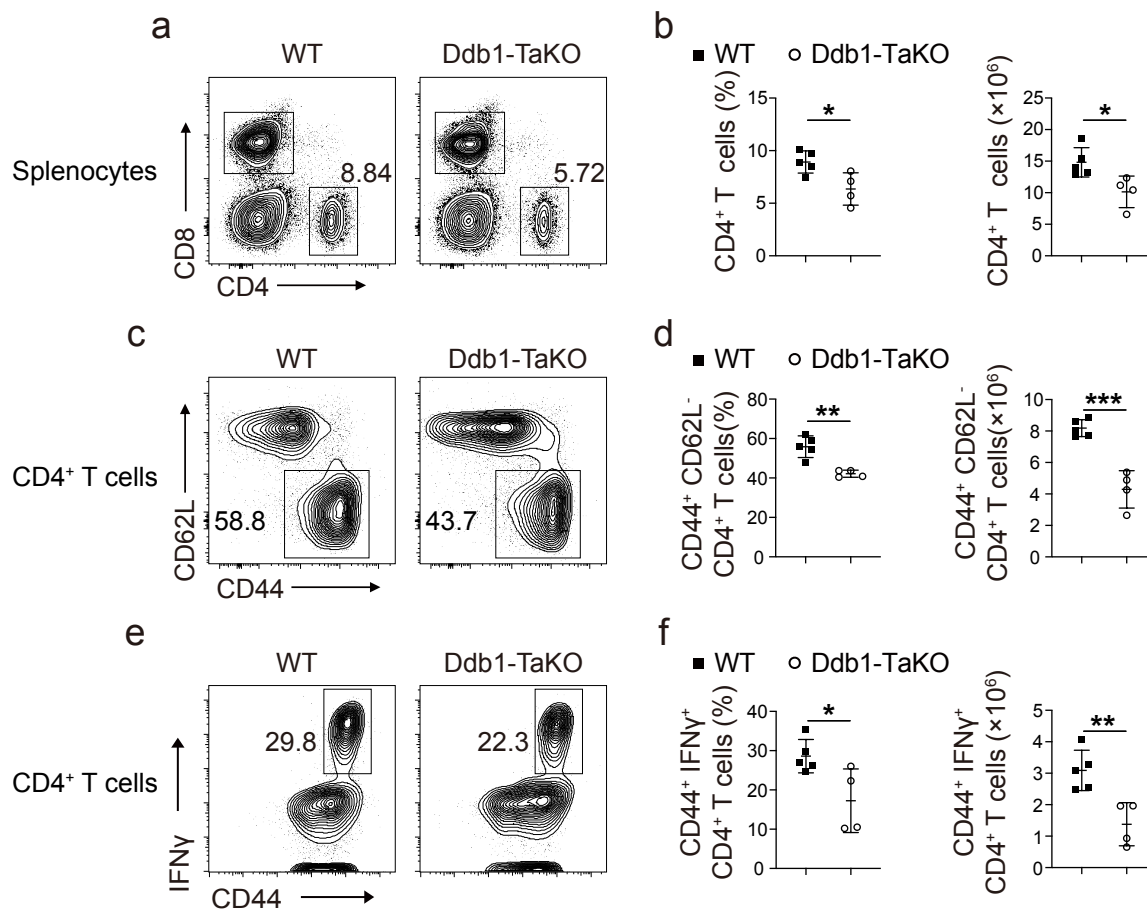

Fig. S5

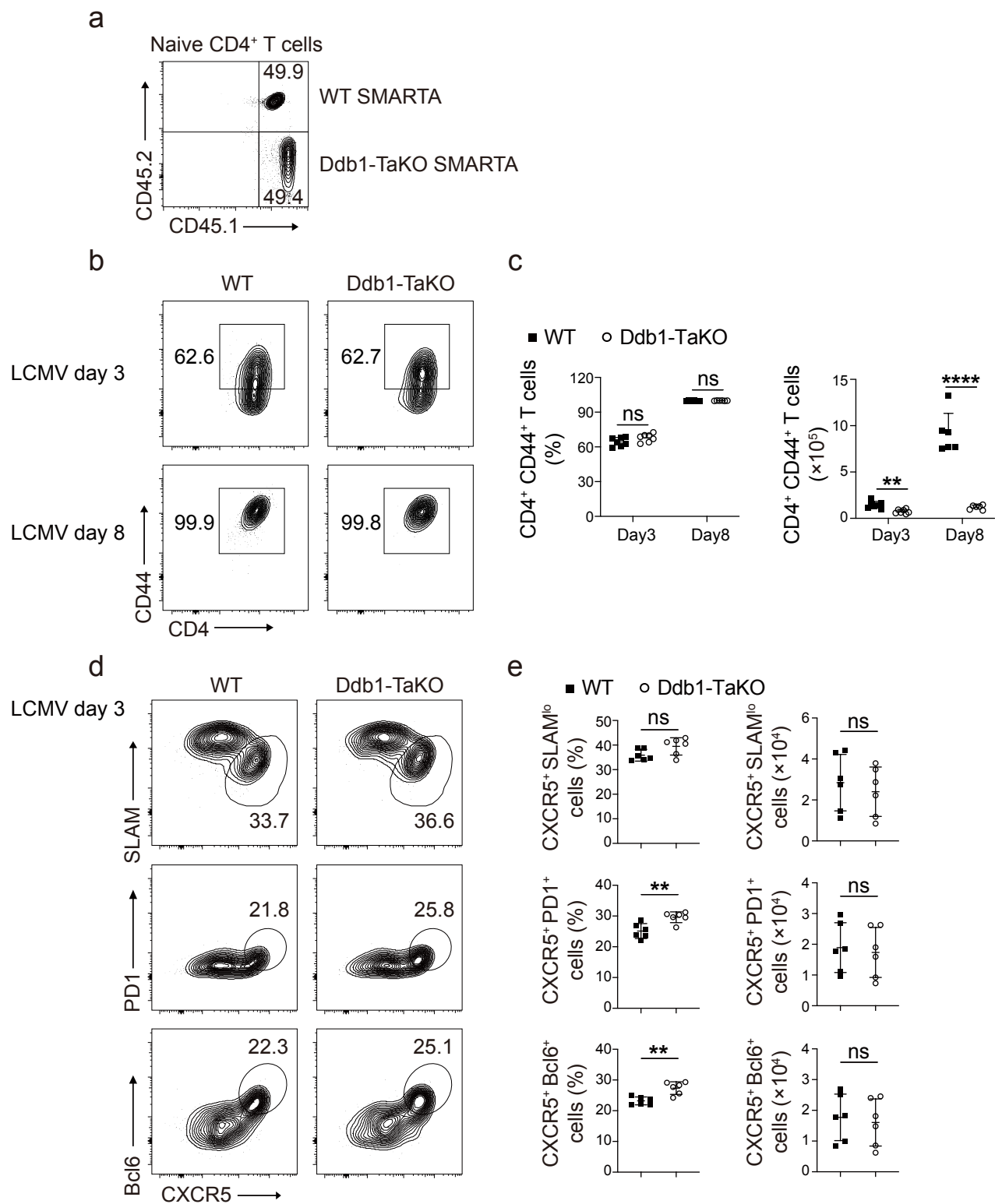

Fig. S6

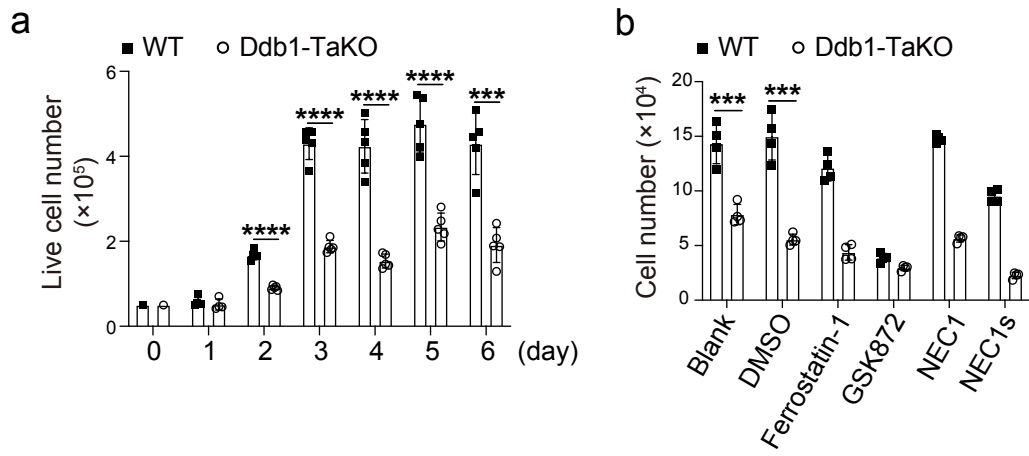

Fig. S7

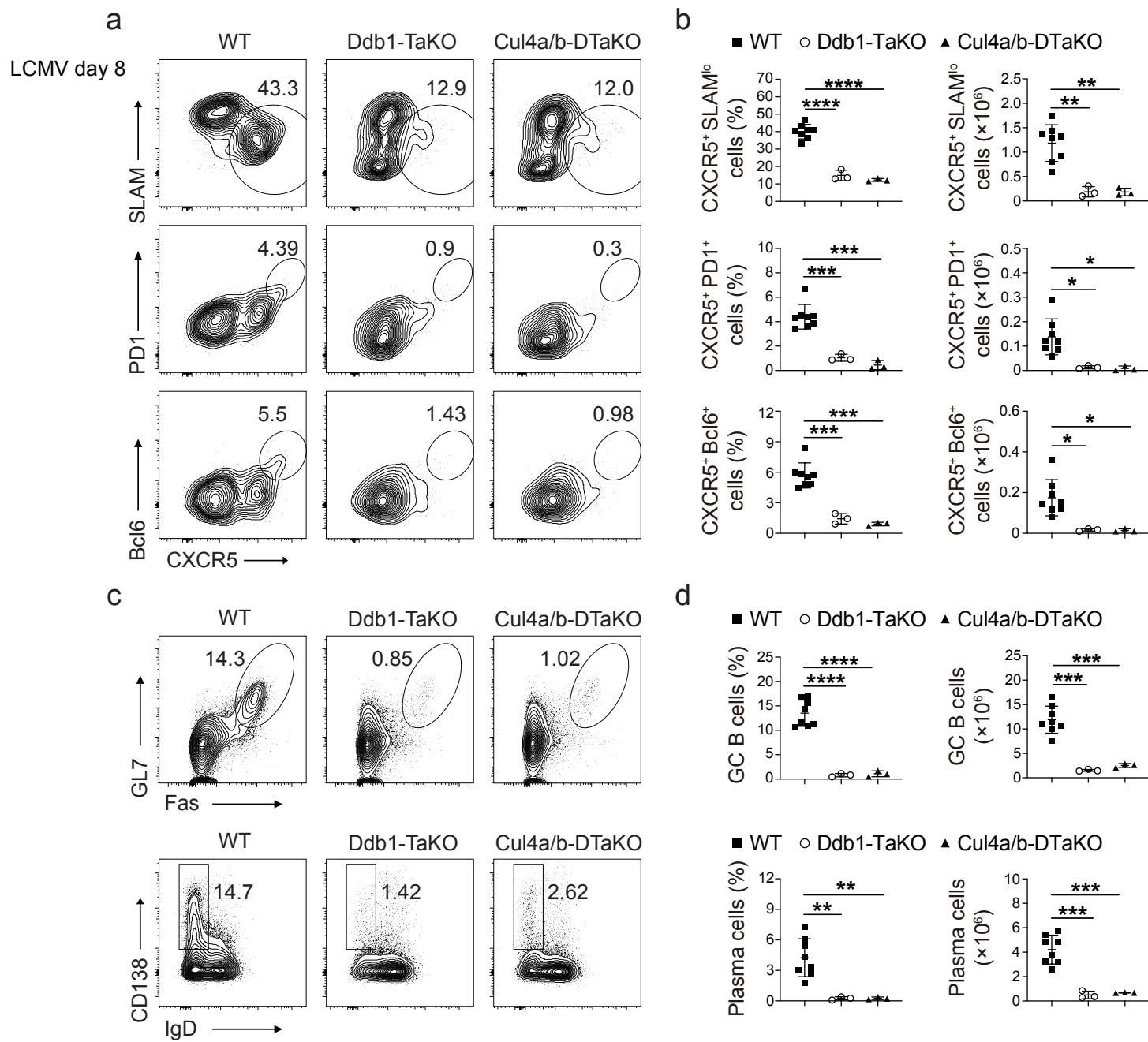

Fig. S8

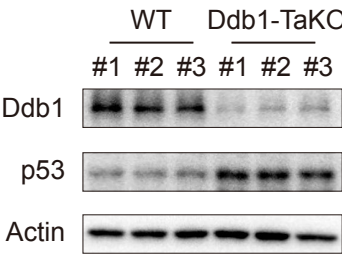

Fig. S9

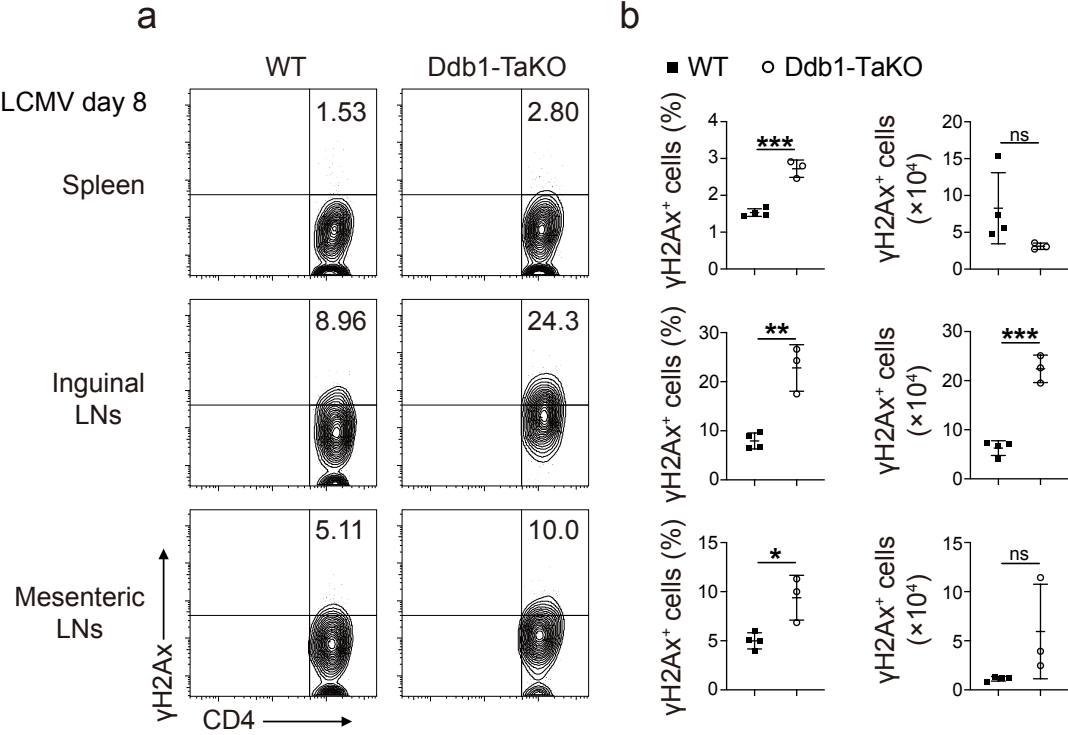

Supplement: Supplementary Figure 1 — The expression of Ddb1 in immune cells and genetic ablation of Ddb1 in activated CD4+ T cells. (A) The mRNA levels of Ddb1 in indicated immune cells (Database: Gene Atlas MOE430, gcrma from BioGPS). (B) Immunoblot analysis of Ddb1 in indicated immune cells. (C) Representative genotyping PCR analysis of tail DNA from WT, Ddb1 fl/+, Ddb1 fl/fl mice displayed on a 2% agarose gel. (D) Immunoblot analysis of Ddb1 in naïve CD4+ T cells purified from WT and Ddb1-TaKO mice and stimulated with 3μg/ml anti-mouse CD3 and anti-mouse CD28 for indicated times. Data are representative of three independent experiments (error bars, s.d.). [file DataSheet_1.pdf]
